# Heart Angiotensin-Converting Enzyme and Angiotensin-Converting Enzyme 2 Gene Expression Associated With Male Sex and Salt-Sensitive Hypertension in the Dahl Rat

**DOI:** 10.3389/fphys.2021.663819

**Published:** 2021-07-19

**Authors:** Kirsti Ytrehus, Stian Ludvigsen, Costantino Mancusi, Eva Gerdts, Giovanni de Simone

**Affiliations:** ^1^Department of Medical Biology, University of Tromsø – The Arctic University of Norway, Tromsø, Norway; ^2^Department of Advanced Biomedical Science, Federico II University of Naples, Naples, Italy; ^3^Department of Clinical Science, University of Bergen, Bergen, Norway; ^4^Department of Heart Disease, Haukeland University Hospital, Bergen, Norway

**Keywords:** hypertensive heart, angiotensin-converting enzyme, male, female, rat, salt-sensitive, fructose, angiotensin-converting enzyme 2

## Abstract

Angiotensin-converting enzyme 2 (ACE 2) in the heart including its sex dependency in the hypertensive heart, has not been much studied compared to ACE. In the present study, we used the Dahl salt-sensitive rat exposed to fructose and salt to model a hypertensive phenotype in males, females, and ovariectomized females. Blood pressure was measured by the tale-cuff technique in the conscious state. Expression of RAS-related genes ACE, ACE2, angiotensin II receptor type 1, Mas1, and CMA1 in the heart were quantified. The results revealed small but significant differences between male and female groups. The main results indicate the presence of a male preponderance for an increase in ACE and ACE2 gene expression. The results are in accordance with the role of androgens or male chromosomal complement in controlling the expression of the two ACE genes.

## Introduction

The COVID-19 pandemic has drawn attention to the connection between cardiovascular disease and coronavirus infections. Hypertension seems to increase the risk of symptomatic COVID-19 infection. Male sex is overrepresented among those with severe disease development and fatality (Borges do Nascimento et al., 2020; Gebhard et al., 2020). Male and female sex hormones are steroids with the regulation of gene expression as their main (but not only) mechanism of action. Sex steroid-responsive elements are present in various cells of the heart.

Angiotensin-converting enzyme 2 (ACE2) (Gheblawi et al., 2020), the cell membrane ectopeptidase linked to the renin-angiotensin system (RAS), is described to function as a receptor for the penetration of the coronavirus into human cells (Chen et al., 2020).

Major RAS components are widely expressed in the body and are important treatment targets against hypertension. Angiotensin II (AngII) produced from angiotensin I (AngI) by the enzyme ACE or chymase, has vasoconstrictive, proinflammatory, and prooxidative effects *via* binding to the AT1R receptor (ACE1/AngII/AT1R axis). An alternative pathway is the conversion of AngI or AngII to the peptide Ang1-7 by the ACE2 enzyme. Ang1-7 binds to a specific MAS receptor promoting anti-fibrotic and vasodilator effects counter-regulating the effect of AngII (ACE2/Ang1-7/Mas axis). Ang1-9 is an alternative product of AngI with an anti-hypertropic effect. At the protein level, ACE is constitutively present in endothelial cells independent of the organ. Proposed cellular sources of ACE2 in the heart are macrophages and in the vessel wall most likely pericytes and cardiomyocytes (Burrell et al., 2005; Chen et al., 2020; Hikmet et al., 2020). The Mas receptor is reported to be present in the sarcolemma of cardiomyocytes (Bader et al., 2018). Chymase in mast cells localized to the heart represents renin and ACE independent pathway to AngII production (Ferrario et al., 2020; Froogh et al., 2020).

The present study aimed to examine if upstream regulation of ACE2 and related RAS gene-expression components in the hypertensive heart were dependent on sex in a female sex hormone-reliant manner. For this purpose, we examined male, female, and ovariectomized female Dahl salt-sensitive hypertensive rats. Only minor differences in gene expression of RAS components were detected. However, male sex but not female sex or loss of ovary function was associated with higher ACH2 and ACE gene expression.

## Method

Six groups (*n* = 14–15) of adult male and female Dahl salt-sensitive rats, aged 12 weeks, were included in the study. The female rats were divided into two groups, one group underwent ovariectomy, and thereafter, the Dahl rats received fructose in their drinking water with a control diet (0.3% NaCl) or an elevated salt diet (6%). The study was approved by local and Norwegian animal welfare authorities (approval ID 6784), and all procedures conformed to the guidelines from Directive 2010/63/EU of the European Parliament on the protection of animals used for scientific purposes. Blood pressure was monitored non-invasively using the tale-cuff technique. After 8 weeks, heart samples from the apex of the left ventricle were harvested and stored in RNA later (Qiagen, Hilden, Germany) and expression of selected target genes ACE, ACE2, Agtr1, MAS1, and CMA1 were examined (Table 1). Total RNA was isolated according to the RNeasyFibrous Tissue protocol (Qiagen). RNA concentration was measured spectrophotometrically (NanoDrop, Witec, Switzerland). Reverse transcription of RNA was carried out using a High-Capacity cDNA Reverse Transcription Kit (Applied Biosystems, Foster City, CA, USA). The qRT-PCR was performed in an ABI PRISM 7900 HT Fast real-time thermal cycler using the SYBR green master mix (Applied Biosystems). Primers were obtained from Eurogentec (Seraing, Belgium) and Sigma-Aldrich (St Louis, Mo, USA). The relative expression ratio of the target gene was calculated using the 2^−ΔΔCT^ method. The expression of the target genes was normalized to stably expressed reference genes succinate dehydrogenase complex flavoprotein subunit A (SDHA) and hypoxanthine-guanine phosphoribosyltransferase (HPRT) selected based on testing by NormFinder (Andersen et al., 2004).

**Table 1:** List of primers for gene expression analysis with their corresponding protein names.

**Gene**	**Protein**		**Primer**
HPRT1 (HPRT)	Hypoxanthine-guanine phosphoribosyltransferase	NM_012583.2	GACCGGTTCTGTCATGTCG
			ACCTGGTTCATCATCACTAATCAC
SDHA	Succinate dehydrogenase complex, subunit A, flavoprotein variant	NM_130428.1	CCCTGAGCATTGCAGAATC
			CATTTGCCTTAATCGGAGGA
Ace (ACE1)	Angiotensin I converting enzyme	NM_012544.1	GGAGACGACTTACAGTGTAGCC
			CACACCCAAAGCAATTCTTC
Ace2	Angiotensin I converting enzyme 2	NM_001012006.1	TCAAGGGAAAAGAACCAGACA
			GGTTTCAAATCACTCACCCATAC
Agtr1	Rattus norvegicus angiotensin II receptor type 1, type 1b (Agtr1b)	NM_031009.2	GGTTCAAAGCCTGCAAGTGAA
			GAGTGAGCTGCTTAGCCCAAA
Cma1 (CYH; MCT1; chymase)	chymase 1, mast cell	NM_013092.1	ACTCTCGGCCAACTTCAACT
			TTCACGTTTGTTCTGCCCCA
Mas1	MAS1 proto-oncogene, G protein-coupled receptor	NM_012757.2	GGAGAGCCTGATTTCCCCTC
			ACAGTGAGCTGGGTGCTTTG

The statistics were based on a 2 × 3 factorial design with two independent variables, diet-salt two levels (with and without salt) and sex three levels (male, female, and ovariectomized female), respectively. Statistical analysis was performed using two-way ANOVA (SigmaStat, Systate Software) for the effect of sex and the effect of increased diet salt and interaction between these two. The Holm-Sidac test was used as a *post-hoc* test to test for significance between the six groups. Results are presented as mean ± SEM.

## Results

At the endpoint, blood pressure in the six groups was elevated above values at 12 weeks in untreated low salt diet Dahl rats (historical controls, mean arterial pressure males 101 ± 3.4 mmHg, females 105 ± 3.2, ovx females 102 ± 4.0, *n* = 6). The added salt diet significantly increased both systolic and diastolic arterial blood pressure above the fructose alone (Table 2). Interestingly, there were no differences in blood pressure between the three subgroups within each of the two diet groups i.e., there were no detectable differences in blood pressure between male, female, and female ovariectomized rats suggesting that any difference in gene expression would not be a direct reflection of differences in blood pressure at the endpoint. However, a significant difference in heart weight relative to tibia length was present between females given elevated salt and females given standard salt.

**Table 2:** Blood pressure, body weight, and heart weight (indexed to tibia length).

	**Male**	**Male salt**	**Female**	**Female salt**	**Female OVX**	**Female OVX salt**
Body weight (g)	407 ± 5^+^	393 ± 7^+^	254 ± 2	260 ± 4	293 ± 8^+^	306 ± 2^+^
Heart/tibia (g/cm)	2.5 ± 0.09^+^	2.6 ± 0.07^+^	1.9 ± 0.04	2.2 ± 0.07^*^	2.1 ± 0.06	2.5 ± 0.06^+^^*^
BP systole (mmHg)	156 ± 4.5	186 ± 6.3^*^	161 ± 7.7	183 ± 6.9^*^	153 ± 4.0	192 ± 6.2^*^
BP diastole (mmHg)	111 ± 4.3	139 ± 6.1^*^	117 ± 7.9	138 ± 6.7^*^	100 ± 3.6	143 ± 7.5^*^

*Mean ± SEM (n = 14–15)*,

* *indicates p < 0.05 vs. the corresponding same-sex group with normal salt*,

+ *indicates p < 0.05 vs. the corresponding female ovary-intact normal diet group*.

**Figure 1:**
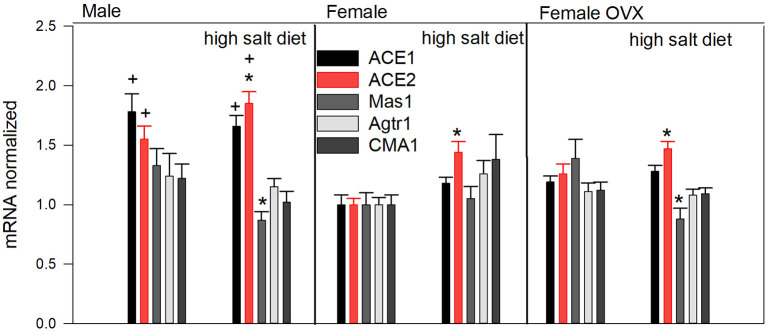
Expression of selected genes (mRNA) normalized to housekeeping genes succinate dehydrogenase complex flavoprotein subunit A (SDHA) and hypoxanthine phosphoribosyltransferase 1 (HPRT1) and presented relative to the expression level in female ovary-intact hearts with standard salt diet. Mean ± SEM (*n* = 14–15), *indicates *p* < 0.05 vs. the corresponding same-sex group with normal salt, ^+^indicates *p* < 0.05 vs. the corresponding female ovary-intact normal diet group (*this group serving as control)*.

Angiotensin-converting enzyme 2 gene expression increased slightly but significantly in all three salt-exposed hypertensive groups (Figure 1). Interestingly, the highest level of ACE2 expression was observed in high salt male hypertensive hearts, which was almost doubled compared to hearts from females with a standard salt diet (1.85 ± 0.1 vs. 1.00 ± 0.05 units). With respect to ACE1, expression was more pronounced in males compared to female hearts, independent of diet intervention. Mas1 tended to be slightly downregulated in hypertensive hearts, however, only significantly in hearts from males and ovariectomized females when compared to corresponding controls.

The gene expression data are presented relative to the expression of the same gene in ovary intact females exposed to normal salt (Figure 1). Normalized to HPRT and STDA measured values were ACE 0.034 ± 0.002, ACE2 0.0032 ± 0.00017, Agtr1α 0.025 ± 0.0016, Mas1 0.0023 ± 0.0024, and CMA1 0.0032 ± 0.0037 (mean ± SEM) in this subgroup. The calculated average ratio ACE2/ACE gene expression was 0.09 ± 0.008 in males, 0.106 ± 0.011 in females, and 0.102 ± 0.007 in female ovx and in corresponding subgroups receiving high salt 0.110 ± 0.008, 0.120 ± 0.009, and 0.112 ± 0.005 (*p* = 0.036, overall effect of the high salt intervention).

## Discussion

Salt-dependent hypertension is widely distributed in the human population and is caused by the interaction between diet and individual genetic makeup (Rodriguez-Iturbe et al., 2007). The condition is mimicked experimentally in the Dahl salt-sensitive rat, which gradually develops hypertension on a standard diet supplemented with fructose and accelerates its development on the high salt diet (Ludvigsen et al., 2018; Lee et al., 2020). In the present study of RAS-related gene expression in Dahl salt-sensitive male and female rat hearts, we observed that overall expression of ACE and ACE2 was higher in males with elevated blood pressure compared to females. Two levels of elevated blood pressure were studied, and the more pronounced hypertension increased ACE2 gene expression slightly but significantly in all three groups. Interestingly, we did not find significant differences between hearts from intact females and ovariectomized females. Thus, the presence of androgens and/or other compounds related to the difference in sex chromosome patterns between males and females might be responsible for slightly higher ACE and ACE2 expression in males in the present study.

Angiotensin-converting enzyme 2 is an X-chromosome linked in both humans and rats. In the case of functionally significant ACE2 polymorphisms in the population, males depend on their maternal variant of the gene, whereas females are mosaic and can be more robust or phenotypically variable. In humans, it is proposed that the ACE2 belongs to a part of the X chromosome that escapes inactivation. To our knowledge, it is not known if this is the case in the rat. In accordance with a connection between androgens and ACE2 expression, ACE2 is present in the male reproductive tract and is especially highly expressed in the testis (Younis et al., 2020). Conclusions from the present study are substantially limited since differences in expression were statistically significant but small, and their physiological relevance appears therefore unclear. Gene expression changes do not predict ACE2 level at the cell surface. We cannot deduce that male hearts have more receptors for the coronavirus; however, we conclude that the regulation at gene expression level by male sex or androgens needs to be examined in different experimental models. Interestingly, the results based on gene expression in the Dahl salt-sensitive rat is in agreement with findings from a study undertaken in spontaneously hypertensive rats measuring ACE and ACE2 activity in male and female hypertensive hearts (Dalpiaz et al., 2015).

Compared to ACE, the ACE2 gene is expressed at a low level, and the overall significance of ACE in the male heart should not be underscored. It has repeatedly been shown that ACE inhibition and AngII receptor antagonism are effective treatments against blood pressure elevation and the progression of concomitant observed heart hypertrophy and remodeling in the Dahl rat (Kim et al., 2001). Heart hypertrophy, fibrosis, and inflammation are the main findings when salt is started at an early age (7 weeks) leading to cardiac failure mirroring heart failure with preserved ejection fraction (HFpEF) after 10 weeks (Gallet et al., 2016). With respect to experimental studies of pressure-induced heart remodeling, most studies are performed in the male. However, in heart failure mast cells, macrophages and T cells contribute significantly and add to the complexity regarding the role of chromosomal sex and sex hormones. This study does not give any evidence to propose a cell source of the various components of RAS in the heart. CMA1 was included since mast cell is part of a hypertensive remodeling of the heart, chymase is a pathway for ACE independent conversion of AngI to AngII in the heart, and sexual dimorphism has been proposed with estrogen as a regulating factor (Li et al., 2015). The decrease in detected expression in high salt male hearts and female ovariectomized hearts is minor, and all hearts studied were obtained from fructose-feed rats with elevated blood pressure (Tran et al., 2009).

In conclusion, the increased expression level of ACE and ACE2 found in the male hypertensive heart indicates that more attention should be paid to mechanisms regulating the different parts of RAS by taking both hormonal status and sex into consideration.

## Data Availability Statement

The original contributions presented in the study are included in the article/supplementary materials, further inquiries can be directed to the corresponding author.

## Ethics Statement

The animal study was reviewed and approved by The Norwegian animal welfare authorities. FDU ID 6784.

## Author Contributions

KY responsible for conception and design of the study, responsible for interpretation of the results, and drafted the manuscript. SL designed and performed the experiments, analyzed the data and drafted figures, and participated in interpretation. CM participated in developing the experimental model, analyzed data, and contributed to preparation of the manuscript as well as interpretation of results. GS and EG were responsible for conception and design of the study as well as interpretation of results. All authors contributed to the article and approved the submitted version.

## Conflict of Interest

The authors declare that the research was conducted in the absence of any commercial or financial relationships that could be construed as a potential conflict of interest.
